# Mounier–Kuhn syndrome: a case of tracheal smooth muscle remodeling

**DOI:** 10.1002/ccr3.794

**Published:** 2016-12-29

**Authors:** Daniel P. Cook, Ryan J. Adam, Mahmoud H. Abou Alaiwa, Michael Eberlein, Julia A. Klesney‐Tait, Kalpaj R. Parekh, David K. Meyerholz, David A. Stoltz

**Affiliations:** ^1^ Department of Internal MedicineUniversity of IowaIowa CityIowaUSA; ^2^ Department of Molecular Physiology and BiophysicsUniversity of IowaIowa CityIowaUSA; ^3^ Department of Biomedical EngineeringUniversity of IowaIowa CityIowaUSA; ^4^ Department of Cardiothoracic SurgeryUniversity of IowaIowa CityIowaUSA; ^5^ Department of PathologyUniversity of IowaIowa CityIowaUSA; ^6^ Pappajohn Biomedical InstituteUniversity of IowaIowa CityIowaUSA

**Keywords:** Airway remodeling, airway smooth muscle, Mounier–Kuhn syndrome, tracheobronchomalacia

## Abstract

Mounier–Kuhn syndrome is a rare clinical disorder characterized by tracheobronchial dilation and recurrent lower respiratory tract infections. While the etiology of the disease remains unknown, histopathological analysis of Mounier–Kuhn airways demonstrates that the disease is, in part, characterized by cellular changes in airway smooth muscle.

## Introduction

Mounier–Kuhn syndrome is a rare clinical entity, with an uncertain cause, recognized by tracheobronchomegaly, airway cartilage ring abnormalities, and lung infections 1, 2, 3. We report a case of Mounier–Kuhn syndrome that we attribute in part to smooth muscle cell remodeling.

## Case Report

**Figure 1:**
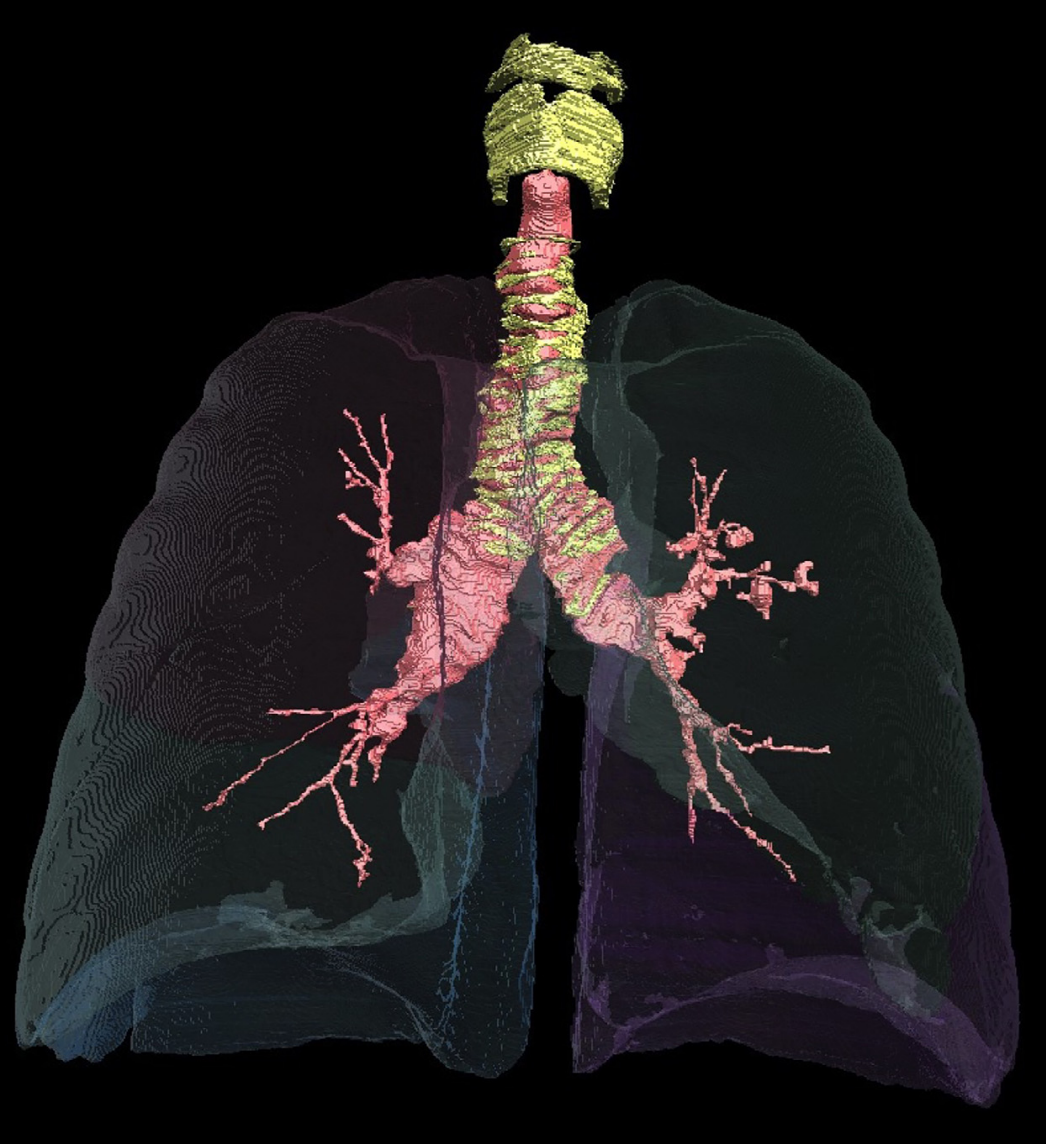
CT‐based 3D reconstruction of airway tree from an adult with Mounier–Kuhn syndrome. Airways are outlined in pink and cartilage in yellow.

A 44‐year‐old man was evaluated for chronic cough, dyspnea, and recurrent lung infections. Pulmonary function tests showed a FEV_1_ of 39% of predicted and sputum culture grew *Staphylococcus aureus*,* Pseudomonas aeruginosa*,* Acinetobacter haemolyticus*, and *Haemophilus parainfluenzae*. An X‐ray computed tomography scan of the chest demonstrated bronchial wall thickening, mucus plugged airways, and bronchiectasis. The proximal airways were dilated (tracheal diameter >3.0 cm) and tortuous with irregular‐appearing cartilage rings (Fig. 1; Video S1). The patient was diagnosed with Mounier–Kuhn syndrome. He experienced progressive airflow obstruction, and thirteen years later, he developed hypoxic/hypercarbic respiratory failure. He underwent bilateral lung transplantation and demonstrated improvements in FEV_1_ percent of predicted 4. However, with time the patient developed respiratory failure and died. At the time of lung transplantation and at autopsy, airway and lung samples were collected which were later used to further investigate potential etiologies of Mounier–Kuhn syndrome and tracheobronchomalacia in this patient.

## Discussion

While the underlying etiology of Mounier–Kuhn syndrome is often attributed to congenital absence or atrophy of airway smooth muscle 2, 3, there has been little focus on airway smooth muscle elements in prior histological studies of patients with Mounier–Kuhn syndrome.

Other potential etiologies of Mounier–Kuhn syndrome have been proposed including the disappearance of the connective tissue network of the airway wall 5, a chronic inflammatory state leading to greater matrix metalloproteinase activity 6, and atrophy of elastic tissues in the trachea and main bronchial walls 7, 8. Moreover, several reports have described an association between Mounier–Kuhn syndrome and connective tissue diseases such as Ehlers–Danlos syndrome, Marfan syndrome, and cutis laxa 9, 10, 11, but an exact genetic etiology remains unknown. The reduction and/or atrophy of the elastic fibers of the tracheal posterior membrane are proposed to contribute to excessive tracheal collapse during expiration 12. This extreme narrowing causes airflow obstruction and subsequent dyspnea, difficulty clearing secretions, and recurrent lung infections. However, the small number of Mounier–Kuhn syndrome cases and scarce histopathological studies 6, 13, 14, 15, 16, 17 limit our ability to determine pathophysiological connections.

**Figure 2:**
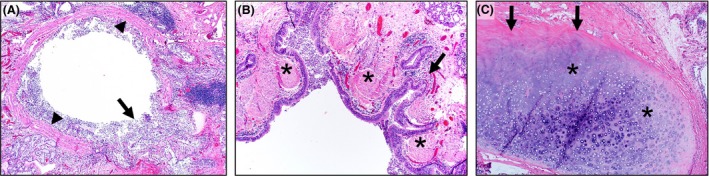
At transplant, histopathology of Mounier–Kuhn lung (HE stain, 40x). (A) A bronchus with circumferential smooth muscle (arrowheads) except for a focal epithelial‐lined pseudodiverticula (arrow) that lacked smooth muscle and had localized remodeling (e.g., fibrosis) of the airway wall. (B) Bronchus with highly folded mucosa. Smooth muscle (asterisks) was seen near the luminal tips, but was lacking near the mucosal recesses (arrow). (C) Bronchial cartilage (asterisks) had localized remodeling along the periphery (arrows).

**Figure 3:**
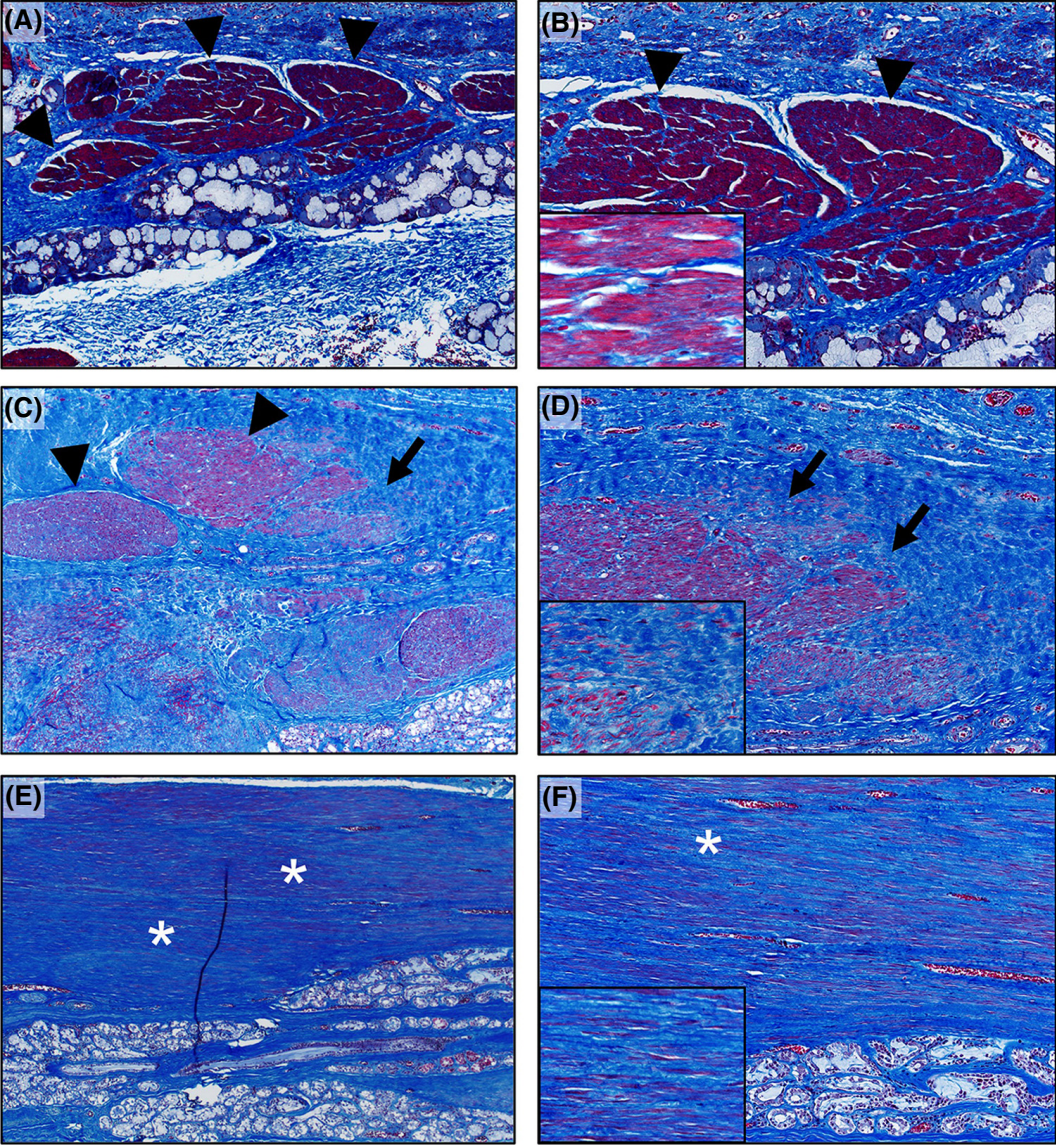
At autopsy, Mounier–Kuhn tracheal histopathology. (A–B) Healthy control tracheal wall with normal‐appearing smooth muscle present (arrowheads). (C–D) Mounier–Kuhn tracheal wall showing smooth muscle (arrowheads) with degeneration and progressive transition (arrows) to fibrosis (blue color). (E–F) Mounier–Kuhn tracheal wall showing extensive fibrosis (asterisks) with scant evidence of smooth muscle. Masson's trichrome stain. 4x (A, C, E); 10x (B, D, F); insets (60x).

By understanding the histopathological changes in Mounier–Kuhn syndrome, we can better define the underlying disease mechanisms. Therefore, we investigated whether airway smooth muscle abnormalities were present in this patient's airways. At lung transplantation, his native lungs had airways with normal‐appearing smooth muscle, but also regions with airway wall degeneration consisting of focal epithelial‐lined pseudodiverticula that lacked smooth muscle and had localized remodeling (Fig. 2A and B). Additionally, bronchial cartilage had localized remodeling along the periphery reminiscent of bony remodeling (Fig. 2C). At autopsy, examination of his native trachea revealed the presence of remodeled smooth muscle with areas of regional degeneration of trachealis muscle and replacement by collagen fibrosis (Fig. 3A–F).

The above histopathological findings in this patient with Mounier–Kuhn syndrome demonstrate that: (i) airway smooth muscle is present, and not congenitally absent; and (ii) regions of airway smooth muscle had undergone degeneration and fibrotic remodeling, as opposed to atrophic loss. Other proposed etiologies for Mounier–Kuhn syndrome 2, 3, including inflammatory and/or elastolysis alterations 6, 17, 18, may be accompanied with progressive airway smooth muscle changes. These observations suggest that tracheobronchomalacia in Mounier–Kuhn syndrome may be, in part, due to localized degeneration/remodeling in airway smooth muscle.

## Conclusions

We report a case of Mounier–Kuhn syndrome with histopathological findings of localized airway smooth muscle degeneration and remodeling. These findings were in addition to widely recognized manifestations of the disease including tracheobronchomegaly and airway cartilage ring abnormalities. Observations in this Mounier–Kuhn syndrome patient suggest that tracheobronchomalacia is not due to congenital absence of airway smooth muscle, but can be associated with localized degeneration/remodeling from unknown etiology(s), which may progress with time.

## Authorship

DPC, DKM, and DAS: performed conception and design of study; DPC, RJA, MHA, ME, JAKT, KRP, DKM, and DAS: performed data acquisition and interpretation; DPC, RJA, DKM, and DAS: prepared figures; DPC, DKM, and DAS: drafted the manuscript; DPC, RJA, MHA, ME, JAKT, KRP, DKM, and DAS: edited, reviewed, and approved final version of the manuscript.

## Conflict of Interest

No conflicts of interest exist for any of the authors.

## Supporting information


**Video S1.** Video of CT‐based 3D reconstruction of airway tree from an adult with Mounier‐Kuhn syndrome. Airways are outlined in pink and cartilage in yellow.
